# Infrared absorbers inspired by nature

**DOI:** 10.1098/rsif.2024.0284

**Published:** 2025-02-19

**Authors:** Sébastien R. Mouchet

**Affiliations:** ^1^ Department of Physics, Namur Institute of Structured Matter (NISM) & Institute of Life, Earth and Environment (ILEE), University of Namur, Rue de Bruxelles 61, Namur 5000, Belgium; ^2^ School of Physics, University of Exeter, Stocker Road, Exeter EX4 4QL, UK

**Keywords:** light absorption, infrared absorber, solar energy, energy efficiency, bioinspiration, photonics

## Abstract

Efficient energy harvesting, conversion and recycling technologies are crucial for addressing the challenges faced by modern societies and the global economy. The potential of harnessing mid-infrared (mid-IR) thermal radiation as a pervasive and readily available energy source has so far not been fully exploited, particularly through bioinspiration. In this article, by reviewing existing photon-based strategies and the efficiency of natural systems in harnessing light and thermal radiation, I highlight the promising role of bioinspiration in enhancing energy capture, conversion and recycling. Natural photonic structures found in various organisms, including insects, birds and plants, exhibit sophisticated optical properties that can be leveraged for energy-efficient applications. These developments pave the way for future research and innovation in bioinspired energy solutions. Ultimately, they contribute to the pursuit of a sustainable and environmentally conscious future by harnessing the beauty of nature’s designs to meet humankind’s energy needs.

## 1. Introduction

Addressing the significant challenges faced by modern global society and the world economy, the advancement of efficient energy harvesting and recycling technologies [1–3] stands as a prominent area of research on a global scale. Mid-infrared (mid-IR) thermal radiation, namely, with a wavelength ranging from 3 to 8 μm, represents a pervasive and readily available energy source. This is not only due to the long illumination of some parts of the Earth by the Sun but also because many machinery, engines and industrial processes dissipate energy in the form of heat radiation, distinct from thermal conduction or convection mechanisms.

While the primary energy source may vary in its environmental impact, the recycling of this ‘wasted’ energy presents a sustainable approach to converting radiative heat losses into diverse forms of energy. Numerous mechanical components found in machinery, engines, industrial processes and even household systems generate mid-IR thermal emissions at moderately elevated temperatures, typically ranging from 150°C to 950°C. These emissions are an intrinsic by-product of the regular functioning of these components and constitute an unavoidable energy loss. The prospect of harnessing this radiative heat loss is compelling, as it offers the opportunity to transform it into electrical power, effectively enabling devices to utilize their own recycled radiative heat loss for enhanced functionality.

Photon-based strategies have already played a crucial role in harnessing solar energy, enhancing the performance of energy conversion devices [4–12]. For instance, devices designed for solar light trapping have effectively increased the efficiency of photovoltaic (PV) cells and thermal photovoltaic (TPV) cells. Similar photonic devices are instrumental in augmenting the efficiency of solar thermal panels, or in energy harvesting for thermoelectric generators (TEG), artificial photosynthesis, and photocatalysis.

In nature, numerous biological organisms have developed highly efficient mechanisms to harness thermal radiation, a crucial adaptation for their survival. Over millions of years of evolution, these natural systems have honed specialized characteristics to maximize their radiation harvesting abilities [13,14]. Consequently, certain structures within their integuments have become increasingly inspiring for the development, design and production of energy-efficient materials [14–17]. Bioinspiration emerges as a powerful and promising strategy in this context.

Natural photonic structures found in various animals, including insects, birds and fish, are examples of effective thermal radiation collectors [15,17,18]. In addition, this type of structure exhibits a diverse array of properties, such as structural colours (resulting from light interference in nanostructures) [13,14,19,20], antireflection features [16,18,21], thermoregulation mechanisms [22–26], light-trapping capabilities [27–30] and enhanced light-extraction methods [31]. These properties emerge from the interaction between radiation and structures composed of biopolymers such as chitin, keratin, collagen or cellulose, sometimes in combination with pores.

The existence of these naturally occurring radiation management systems challenges the human imagination. While human beings have access to a wide range of materials, human designs sometimes fall short in complexity compared with these remarkable natural structures. Identifying and comprehending these natural photonic devices not only expands human understanding but also empowers engineers and materials scientists to conceptualize new ideas and explore potential technological applications through bioinspired principles [14–17]. These exciting possibilities have captivated the attention of researchers worldwide. Despite the development of artificial intelligence, bioinspiration remains a guiding force in the quest for novel technological applications. The convergence of both approaches holds promise for unprecedented advancements in this field.

In this article, I first review previously investigated cases of photonic structures enhancing electromagnetic-wave absorption (also known as structural absorption) in natural organisms across the ultraviolet (UV), visible and infrared (IR) range. This is because the dimensions of a visible light absorber occurring in nature may be adjusted to another range such as IR through a bioinspiration approach due to the scalability of Maxwell’s equations.^1^ Finally, I review examples of bioinspired IR absorbers from the literature.

## 2. Light absorption enhanced by photonic structures in natural organisms

The management of electromagnetic radiation and thermoregulation are pivotal functions essential for the survival or benefit of various natural organisms, including plants, insects and birds [23–26,32–37], whether endotherms (organisms able to maintain their body temperature through their metabolisms), mesotherms (organisms with some metabolic strategies of heat production without any proper metabolic heat control) or ectotherms (organisms requiring external heat sources) [38]. For instance, photosynthesis implies absorbing visible radiation from the Sun, whereas thermoregulation of ectothermic animals involves a subtle trade-off between radiation absorption and thermal emission in the near-infrared (near-IR) part of the electromagnetic spectrum. Photonic structures may play roles in the management of such thermal radiation. For instance, iridescent butterflies were reported to exhibit in general an absorptance^2^ higher than that of non-iridescent species [39]. Other striking illustrations are the photonic structures occurring in the super-black feathers of the bird of paradise (as depicted in figure 7) [36], as well as in the scales covering the black wings of insects like the Magellan birdwing and the Meander prepona butterflies [23,27]. These feathers and wings exhibit remarkably high energy absorption properties within the spectral range of solar irradiance, encompassing the mid-IR spectrum in some instances. Often, in such natural integument, incident light is absorbed by pigments including melanin [40–43]. Nanostructures, operating at micro- and nano-metre length scales, yield fascinating opportunities for both light and thermal radiation harvesting.

### 2.1. Wings of lepidopterans

**Figure 1.**
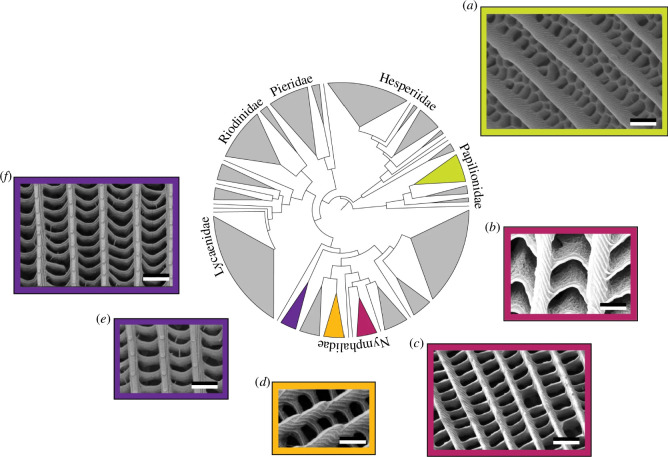
Diverse structures of scales exhibit ultra-black coloration within the order Lepidoptera. They typically comprise holes located in between the scales' ridges with various sizes and shapes: honeycomb (*a*), chevrons (*b*) and rectangles (*c–f*), as observed by scanning electron microscopy (SEM) with wings of *Trogonoptera brookiana* male papilionid (*a*), *Eunica chlorocroa* nymphalid (*b*), *Catonephele antinoe* nymphalid (*c*), *Heliconius doris* nymphalid (*d*), *Euploea dufresne* nymphalid (*e*) and *Euploea klugi* nymphalid (*f*). Scale bars: 1 μm (*a–f*). This figure was reproduced from [44], License CC-BY-4.0.

Insects such as lepidopterans, the taxonomic order encompassing the ethereal beauty of butterflies and the enigmatic allure of moths, are typically ectotherms, which means that their metabolisms rely on environmental heat sources. Harvesting incident energy appears crucial for these species. The phylogenetic diversity of structures giving rise to ultra-black coloration occurring in the order Lepidoptera was recently analysed in detail (figure 1) [44]. All these structures present some longitudinal ridges connected by cross ribs in the upper lamina of the scales, forming two-dimensional networks of quasi-periodic holes. The resulting high surface area was described as increasing light absorption by underlying cuticular melanin and reducing reflection [44,45]: whatever the size and shape of the holes—honeycomb, chevrons or rectangles—scales giving rise to ultra-black visual appearances exhibit steeper ridges as well as deeper and wider trabeculae (namely, pillars connecting the upper and basal laminae of a scale) than scales with some regular black or brown colour. Through numerical modelling, these features were shown to play a significant role in reducing light reflection [44,45]. Furthermore, coating these structures with gold does not lead to an increase in light reflectance, unlike regular black or brown butterfly wings. This experimentally demonstrates the photonic origin of the related light harvesting [44].

**Figure 2.**
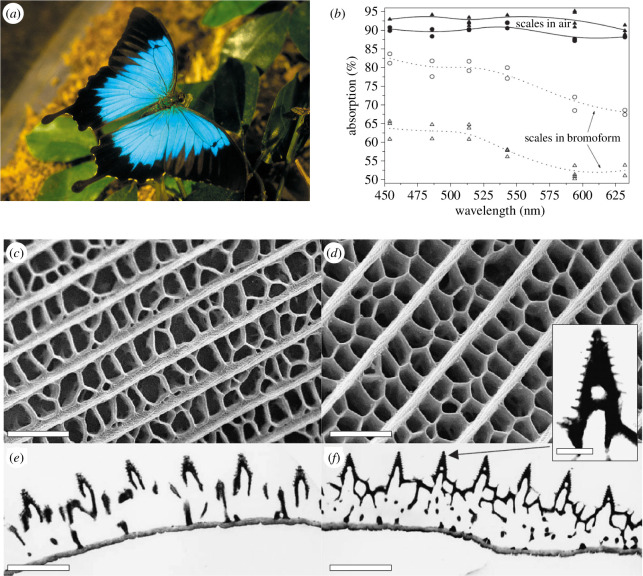
The male *Papilio ulysses* butterfly (*a*) exhibits some black areas on its wings. Upon contact with index-matching fluid (here, bromoform), the absorption spectra exhibit significantly lower intensities (*b*). They were measured at normal incidence with both lustrous (circles) and matt (triangles) black scales. Electron microscopy (*c*,*d*: SEM; *e*,*f*: TEM) allowed the observation of the structures of the lustrous (*c,e*) and matt (*d,f*) black scales. Scale bars: 3 μm (*c*), 2 μm (*d*); 2 μm (*e,f*); inset, 300 nm. These figures were reproduced from W. van Aken (*a*), https://commons.wikimedia.org/wiki/File:CSIRO_ScienceImage_3831_Ulysses_Butterfly.jpg, License CC-BY-3.0, and from [28] (*b–f*), with permission from the Royal Society.

In the scales covering the wings of the male *Papilio ulysses* butterfly, similar complex photonic structures were found to play a role in intensifying the dark areas [28], in addition to the porous multi-layer structure producing the bright blue colour through interference [46]. They were described as scattering light towards the ridges and the interior of the scale, leading to a longer optical path, resulting in a higher light absorption by the pigments distributed within the scale material. For matt black scales, the absorption reduced from 95% to 55% upon contact with bromoform serving as index-matching fluid, while for lustrous black scales, it decreased from 90% to 70% (figure 2). Ridges were, however, demonstrated through simulation to have a neglected role in visible-light absorption of the ultra-black scales of *Pachliopta aristolochiae* [47].

**Figure 3.**
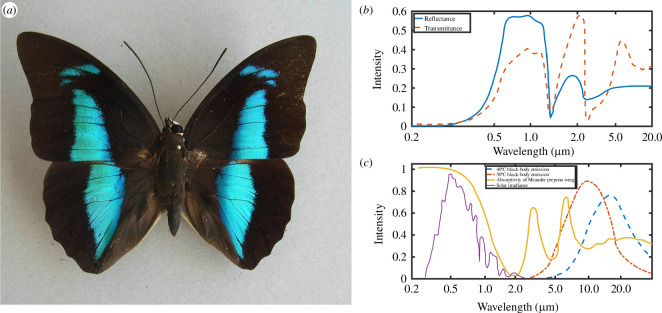
Radiative cooling plays a key role in the thermoregulation of certain butterfly wings, exemplified by *Archaeoprepona meander*, the Meander prepona (*a*). It arises from the morphology of the scales covering the black area of the wings. A clear difference in reflectance and transmittance was measured between the near-IR and visible parts of the electromagnetic spectrum (*b*). Black-body emission spectra at 40°C and 50°C, solar irradiance, and the absorptivity of a wing (*c*) allowed Berthier [23] to explain the thermoregulation enabled by the C=O absorption peak of chitin at 6 μm. Figure (*a*) was reproduced from Notafly, https://commons.wikimedia.org/wiki/File:Archaeoprepona_meander_(Cramer,_-1775-).JPG, License CC-BY-SA-3.0. Data in figures (*b,c*) are from [23].

The wings of *Troides magellanus* butterfly, the Magellan birdwing, present a captivating spectacle of optical properties, including light diffraction and controlled fluorescence emission on their hindwings [48–51]. It inhabits the Philippines and Taiwan’s Orchid Island. Renowned for their impressive size and striking appearance, the forewings showcase a remarkable 98% absorption of visible light as well as reveal two distinctive peaks in the IR spectrum [27]. As detailed in the following paragraph and figure 3, the presence of chitin imparts the wings with these robust absorption peaks at 3 and 6 μm due to C=O vibrations, strategically positioned within the wavelength range where a black body emits radiation at 40°C, enabling radiative cooling.^3^ The architecture of the Magellan birdwing consists of five major elements [27]: a roof-like structure on which a series of ridges are located; holes in the so-called ‘spacer’ structures separating the ridges; and pillars joining the upper membrane to the lower membrane of the wing. A similar structure was also found in the case of the related *Troides aeacus* [52]. Comparison of numerical simulations between the photonic structure and a non-structured flat slab with an equal volume of material showed a 10% increase in electromagnetic radiation absorption and a 17% increase in emissivity at 40°C [27]. This unique combination of optical characteristics suggests that the Magellan birdwing has evolved to manage efficiently both visible and IR light, underscoring the sophisticated adaptation of these butterfly wings for light and thermal radiation purposes.

A related thermoregulation was demonstrated in the case of *Archaeoprepona meander*, the Meander prepona, a tropical butterfly species (figure 3) [23], as well as later on in various butterfly species [53]. These insects employ a sophisticated mechanism to manage their body temperature effectively within a given range such as 36–40°C [23] or 20–50°C [53], depending on the species. The intricate structure of their wings such as the black wings of Meander prepona serves as a remarkable example of nature’s engineering prowess. The scale structures on the wings, in addition to melanin pigments, play a crucial role in harnessing solar energy efficiently, absorbing approximately 95% of the visible solar spectrum (figure 3*c*) [23], akin to the phenomenon described in the cases of *Papilio ulysses* and the Magellan birdwing hereabove. In the near-IR range, the absorptance intensity decreases down to less than 2%, ensuring low thermal emissivity, apart from the absorptance peaks at 3 and 6 μm. The 6 μm emissivity peak plays a crucial role in thermoregulation [23]. At temperatures below 40°C, the black-body peak is located at a longer wavelength (figure 3*c*). It allows the wing to harvest heat effectively while maintaining low thermal emissions. When temperatures exceed 40°C, a significant overlap between the black-body spectrum and the 6 μm peak appears (figure 3*c*), leading to higher thermal emission (namely, radiative cooling) and contributing to the butterfly’s fine-tuned response to thermal challenges in its habitat. This thermoregulation mechanism could be employed in applications such as solar energy harvesting as it can help maintain the devices within an optimal temperature range.

The role of ultra-black colours in butterflies remains the subject of speculation. However, it was hypothesized that they enhance the contrast in the visual signals, as ultra-black areas are always located next to bright areas [44]. Such visual contrast would have implications in terms of aposematism or intraspecific communication.

**Figure 4.**
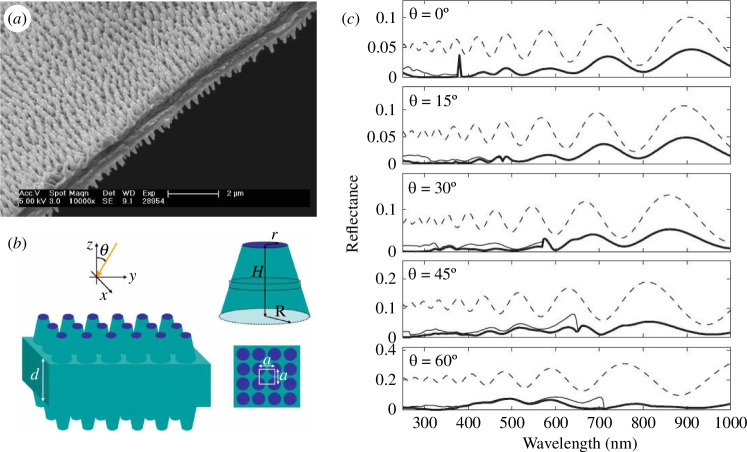
*Cacostatia ossa* moth’s wings exhibit increased transparency due to a unique disordered structure in the nipple array covering them (*a*). This structure was modelled by truncated cones for reflectance simulations (*b*). Its total (thin solid curves) and specular (thick solid curves) reflectance spectra (*c*) at different angles of incidence are much lower than the specular reflectance of an unstructured flat wing (dashed curves). These figures were reproduced from [57], with permission from the American Physical Society.

In addition to structured scales covering the ventral and dorsal sides of lepidopteran wings, many species including butterflies *Greta* spp., the moth *Cacostatia ossa*, and the moth *Cephonodes hylas* display some highly transparent scale-less wings with antireflection properties through photonic structuring of the wing membranes [16,18,54–60]. This structuring curtails reflection of incident light to levels below 2% across the entire visible spectrum through electromagnetic impedance matching. It consists of a lattice of dome-shaped protuberances, also known as nipples (figure 4). The underlying principle behind this antireflection effect lies in the gradual refractive-index matching^4^ between the air and the wing membrane, typically composed of chitin. If the protuberances are spaced by less than the incident wavelength, typically less than 200 nm, the non-zero diffraction orders are evanescent. The protuberance structure can be regarded as a slow variation of the effective refractive index along the normal to the wing membrane. Depending on the species, the protuberance lattice can be very well ordered such as the hexagonal compact array in the wings of *C. hylas* [18,54,55] and *Hemaris fuciformis* [56,60] hawkmoths or more disordered such as the wings of *C. ossa* moth (figure 4) [16,57] and the ones of *Greta* spp. glasswing butterflies [58,59]. Interestingly, a disorder in the protuberance height, width and position was found to increase the transparency properties in the case of *G. oto* glasswing butterfly [59]. Beyond the order of lepidopterans, antireflection structures manifest in the wings of odonatans, such as *Aeshna cyanea* dragonfly [60,61], the American rubyspot damselfly *Hetaerina americana* [58] and *Vestalis amabilis* damselfly [60], or even in hemipterans like cicadas [16,60,62–65]. In addition, such nipple arrays were observed on the surfaces of compound-eye corneas of several arthropods [21,56,66–68]. They are often referred to as moth-eye structures. A comparative study of 19 species of butterflies led to the classification of the arrays into three categories according to their morphologies: conical, paraboloidal and Gaussian. The paraboloid profile with protuberances almost touching each other was found to exhibit the lowest reflectance, with the effective refractive index varying quasi-linearly with depth [21]. Highly antireflective wings in insects are often reported to play a likely role in crypsis [63,64]. Similarly, nipple arrays on the surfaces of compound eyes are assumed to improve camouflage under daylight and improve night vision [66,67]. In general, some of such lattices of protuberances were reported to combine antireflection with hydrophobic properties [16,63,64,69–72], bactericidal activity [72–76] and fluorescence emission [60,77–80]. In the case of cicadas, it was shown that the protrusions could be approximated by truncated cones under hemispheres [16,64]. The cones gave rise to impedance matching and high antireflection, whereas the cones favoured hydrophobicity. Fluorescence arises from fluorescent proteins—typically resilin—embedded within the membrane material [60,77–80].

### 2.2. Elytra of beetles

**Figure 5.**
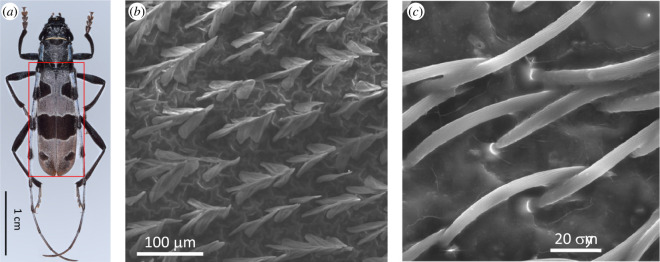
The elytra of *Rosalia alpina* longhorn beetle exhibits black patches on a blue-grey background (*a*). The black patches are covered by so-called ‘tent-shaped’ scales (*b*), whereas hairs cover the blue-grey area (*c*). These figures were reproduced from [81], with permission from Elsevier.

The blue-grey elytra of *Rosalia alpina* longhorn beetle (family Cerambycidae) exhibit large black spots (figure 5). The micro- and nano-structured setae that cover these elytra contribute, on one side, to the camouflage of this beetle on beech barks and, on the other side, to thermoregulation by allowing quick heating of the body to the optimal temperature and dissipating excess heat through IR emission to prevent overheating [81,82], akin to the wings of some butterflies described in the previous section. The setae occurring in the black spots enhance visible-light absorption by light trapping, whereas the setae of all the elytra enable thermoregulation. The former are inclined scales, touching neighbours at the tips and forming tent-like architectures with 1 μm period and 100 nm period grating patterns (figure 5*a*) [81,82]. The setae occurring on the blue-grey areas consist of hairs [81,82] (figure 5*b*). Through optical modelling based on scanning electron microscopy (SEM) observations, the light-trapping role of the scales was demonstrated [81,82]. Several reflections on opposite inclined patterned scales and high concentrations of melanin in these scales account for the high-absorption properties. In addition, the scales and the hairs exhibit absorption (and hence emission) enhancement in the mid-IR range [81,82].

More recently, Vasiljević and co-workers unveiled a combination of lenslets and micrometre-sized multi-layered spherical black elements located within the elytra of the *Morimus asper funereus* longhorn beetle (family Cerambycidae) [83], which also display black spots on a grey surface. However, in this case, both areas, black and grey, look identical when observed with a thermal camera. The authors concluded from finite element method (FEM) simulations that the combined action of the lenslets and the multi-layered spherical elements focuses IR radiation on microchannels containing haemolymph.

**Figure 6.**
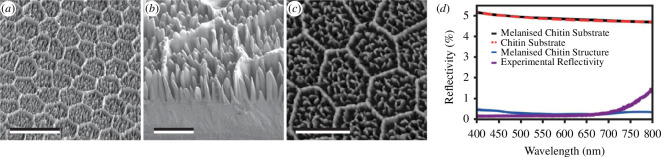
The microstructure occurring on the elytra of *Euprotaetia inexpectata* scarab beetle consists of some ellipsoidal and randomly positioned micropillars (*a–c*) observed here by SEM with different viewing angles. (*d*) Numerical predictions of the spectral reflectance from a flat absorbing chitin substrate (black solid line), a flat non-absorbing chitin substrate (red dashed line) and the described absorbing microstructure (blue solid line) confirm the role of the observed structure in the absorption enhancement. The latter simulations agree with experimental results (purple solid line). Scale bars: 15 μm (*a*), 5 μm (*b*) and 10 μm (*c*). These figures were reproduced from [84], License CC-BY-4.0.

Finally, arrays of ellipsoidal and randomly located micropillars (figure 6*a*–c) were reported on the elytra of *Euprotaetia inexpectata* scarab beetle (family Scarabaeidae) [84]. They enhance light absorption by a combination of Mie scattering and optical focusing. This way, incident light reaches absorbing pigment—namely, melanin—located within the elytra, giving rise to an absorptance up to 99.5% and a reflectance of 0.1% at 400 nm (figure 6*d*).

### 2.3. Bird feathers

**Figure 7.**
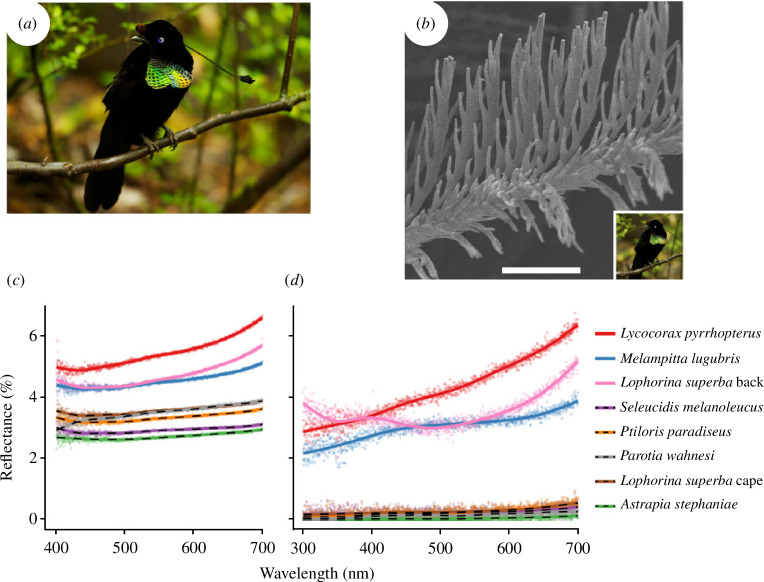
The ultra-black feathers of the *Parotia wahnesi* bird of paradise (*a*) are characterized by specialized barbule arrays, as observed by SEM (*b*). Reflectance spectra (*c,d*) of standard black (solid lines) and ultra-black (dotted lines) feathers are compared, showcasing total (namely, the sum of diffuse and specular components) reflectance (*c*) as well as specular reflectance at normal incidence (*d*). Scale bar: 50 μm (*b*). These figures were reproduced from [36], License CC-BY-4.0.

The ultra-black plumage occurring in some species of birds of paradise within the family Paradisaeidae has captivated researchers due to its unparalleled darkness, reaching absorption levels of up to 99.95%. This phenomenon, elucidated by McCoy and colleagues, is a result of structural absorption rather than pigmentation (figure 7) [36]. These feathers appear even darker than typical black feathers due to a significant reduction in specular reflection, as measured through directional reflectance ranging from a mere 0.05% to 0.31%. The secret lies in the microstructure of the feathers, featuring barbules curved up that are tilted vertically by *ca* 30° with respect to the normal, in the direction of the feathers’ distal tip. This unique arrangement enhances multiple light scattering, creating regularly spaced cavities with dimensions of 5−30 μm in width and 200−400 μm in depth. Astonishingly, the super-black effect is most pronounced when looked at from the distal direction, aligning perfectly with the perspective of a female observing a male. The cavities present a directional reflectance bias, making the feathers even darker when viewed from the distal direction. This natural adaptation showcases the fascinating ways in which birds of paradise have evolved to achieve remarkable visual effects in their plumage.

### 2.4. Cuticle of *Maratus* jumping spiders

**Figure 8.**
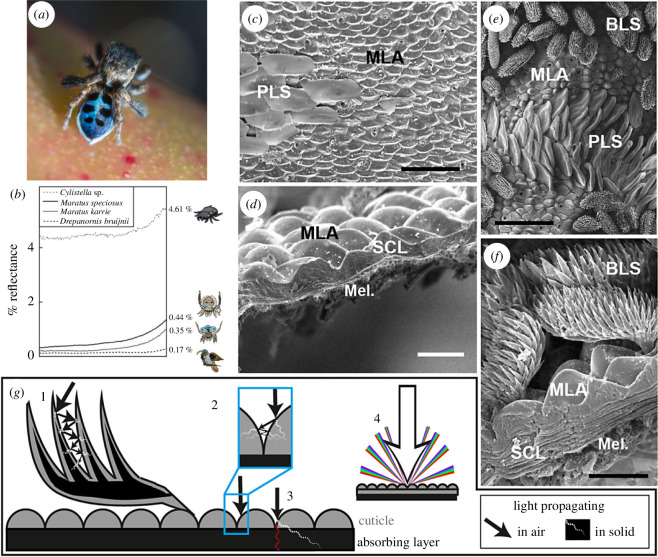
Some male jumping spiders such as *Maratus nigromaculatus* exhibit ultra-black areas along with striking colour for courtship (*a*). These ultra-black areas reflect 0.44 and 0.35% of light with a 30° detection angle (*b*) in the cases of *Maratus speciosus* and *Maratus karrie*, respectively. This is significantly less than the light reflection from the cuticle of a standard black spider such as *Cylistella* sp. (namely, 4.61%) and more than from the black feathers of *Drepanornis bruijnii* bird of paradise (i.e. 0.17%). The ultra-blackness of spiders such as *M. speciosus* arises from microlens arrays (MLA) covering some striated layers in the cuticle (SLC) and some absorbing layer of melanin (Mel.) observed in (*c,d*). Some plate-like scales (PLS) giving rise to blue colour are also imaged in (*c*). In the case of *M. karrie* (*e,f*), some additional brush-like scales (BLS) occur on the surface of the integument. They enhance further light absorption. McCoy and co-workers [85] described four mechanisms of light absorption (*g*): (ii) light is scattered multiple times as it interacts with spiny projections of the BLS, gradually being absorbed as it traverses through the cuticle material and into the absorbing melanin layer while scattering; (ii) multiple scatterings occur between bump surfaces, causing light to be absorbed as it passes through the cuticle materials and enters the melanin layer; (iii) the path length of light within melanin layers is extended, leading to increased absorption; and (iv) light undergoes diffraction due to a periodic microlens array, resulting in reduced visual detection by an observer such as a female spider. Scale bars: 30 μm (*c*), 10 μm (*d*), 50 μm (*e*) and 10 μm (*f*). These figures were reproduced from Graham Wise (*a*), https://commons.wikimedia.org/wiki/File:Maratus_nigromaculatus_(14585680722).jpg, License CC-BY−2.0 and from [85] (*b-g*), License CC-BY-4.0.

Jumping spiders, specifically the male members of the genus *Maratus*, commonly referred to as peacock spiders, have evolved a fascinating display strategy to attract their female counterparts (figure 8*a*). These spiders exhibit a striking combination of brilliant colours arising from pigments or photonic structures [86] and velvety black areas [85] on their bodies. These black regions, described as ultra-black [85], reflect less than 0.5% of light, reaching intensities as low as 0.35% in the case of *Maratus karrie* due to microstructures, including densely packed cuticular bumps resembling microlens arrays (figure 8*c*,*d*). In addition, *M. karrie* displays some black scales resembling brushes (figure 8*e*,*f*). Optical modelling revealed a delicate balance between minimizing light reflection from the surface and maximizing absorption by melanin (figure 8*g*). Interestingly, McCoy and co-workers proposed that this ultra-black followed a convergent evolution for the success of these spiders and the birds of paradise in the competitive realm of sexual selection [85].

### 2.5. Skin of West African Gaboon viper

**Figure 9.**
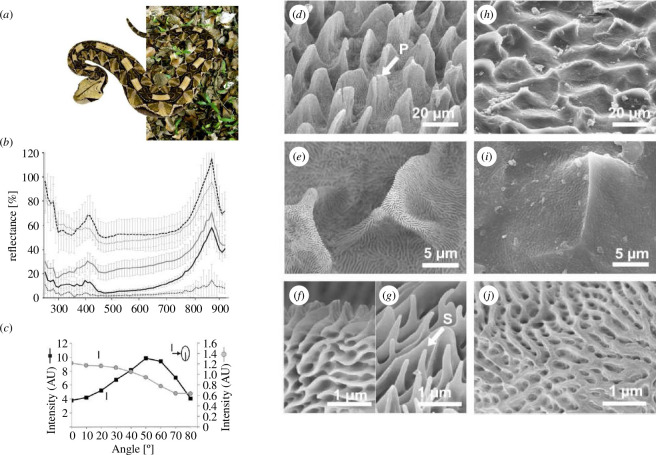
The West African Gaboon viper, *Bitis rhinoceros*, (*a*) exhibits an effective camouflage pattern with velvety black, light brown and pale hue areas on its skin. Reflectance spectra (*b*) highlight the characteristics of black dorsal scales (solid black line), Au–Pd coated black dorsal scales (dotted black line), pale dorsal scales (solid grey line), Au–Pd coated pale dorsal scales (dotted grey line) and ventral scales (dashed black line). Unlike pale scales (solid black line), black scales (solid grey line) do not specularly reflect light (*c*): with a 700 nm incident light at 45° with respect to the normal to the skin surface, light reflectance decreases with the detection angle (*c*). The microstructures of the scales can be imaged by SEM (*d–j*). The black scales are densely packed and resemble leaves marked with an arrow (P) in (*d*). They are covered with small ridges (*e–g*) with spinules indicated with an arrow (S) in (*g*). The pale scales have a more simple pattern (*h*), exhibiting pits (*i,j*). These figures were reproduced from [87], with permission from Springer Nature.

The Gaboon viper *Bitis rhinoceros*, native to West Africa, exhibits a stunning camouflage in its natural habitat, thanks to its intricate skin pattern [87]. The geometrically arranged velvet black spots, interspersed with pale and light brown regions (figure 9), seamlessly blend into the diverse light and shade patterns of the forest ground under the canopy. Observations revealed that the blackness of the viper’s scales is primarily derived from a hierarchical structure characterized by densely packed, leaf-like microstructures covered with nanoridges. Under microscopic scrutiny, even the areas in between black scales exhibit nanoridge striations (figure 9). Reflectance spectra analysis demonstrates that both black and pale scales have nearly flat profiles across the visible range, with a notable peak around 880 nm (figure 9). Intriguingly, applying an Au–Pd coating to the black scales preserves the black colour and further diminishes reflectance (figure 9). This finding supports the idea that the viper’s original surface works as an effective light-trapping device, utilizing multiple reflections of light. The metallic coating further enhances light trapping via light reflections on the metal-coated surfaces. Modelling of diffuse reflection using Lambertian symmetric V-shaped cavities validated the proposed light-trapping mechanism and elucidated the angular dependence of reflectance spectra in pale scales [87]. However, the black scales exhibit a distinct angular characteristic, lacking a specular reflection peak and displaying a gradual decrease in reflectance intensity with increasing emerging angles. This unique angular behaviour imparts a non-glossy visual appearance to the velvet black, attributed to the more isotropic arrangement of scale structure.

### 2.6. Plants and algae

**Figure 10.**
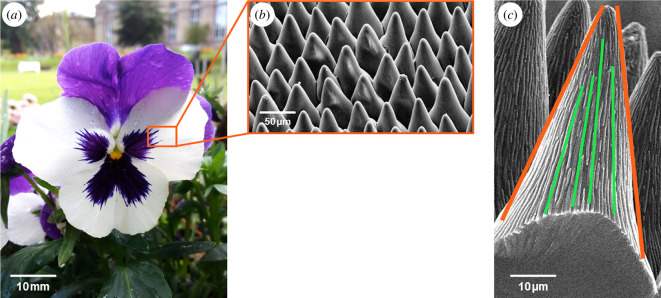
The petals of viola flowers (*a*) feature cone-shaped structures adorned with nanowrinkles (*b,c*). The cones and nanowrinkles collectively play a role in the augmentation of light harvesting. These figures were reproduced from [88], with permission from the American Chemical Society.

In the realm of plants, the surfaces of some petals and leaves reveal a mesmerising array of structures optimized to enhance light harvesting (figure 10). The interplay of antireflection and light trapping mechanisms unfolds through the subtle architecture of conical-shaped epidermal cells [34,88–90]. As sunlight encounters these structures, a gradual increase in the effective refractive index occurs, giving rise to antireflection akin to the cones found on the surfaces of moth eyes and cicada wings. In some plants, additional nanowrinkles were demonstrated to reduce light reflection [34,88]. In addition to antireflection, light redirection extends the path length within plant integuments, contributing to light trapping. The epidermal cells of flowers were reported to function as lenses and conduct incident light into the integuments comprising pigments [89,91,92]. It was also shown that the cone shape of their petals varies, reflecting the plant’s strategic adaptation to either scatter or absorb incident tUV waves, with shorter cones in the former, and taller ones in the latter, respectively [93]. This adaptive variability plays a pivotal role in enhancing light capture for crucial processes such as photosynthesis and contributes to the vivid coloration of these botanical wonders, especially in environments with limited light availability.

Venturing into the microscopic world, diatoms, unicellular algae encased in intricate silica frustules (namely, the hard porous structures of diatoms), offer a different yet equally captivating story of solar energy harvesting [94]. The case of *Coscinodiscus* sp. stands out, with its frustule comprising three layers—termed cribellum, cribrum and the internal plate—each composed of thin slabs housing hexagonal arrays of disk holes. The size and spacing of these holes vary from layer to layer, forming a hierarchical structure that has been finely tuned for optimal light trapping and photosynthesis.

**Figure 11.**
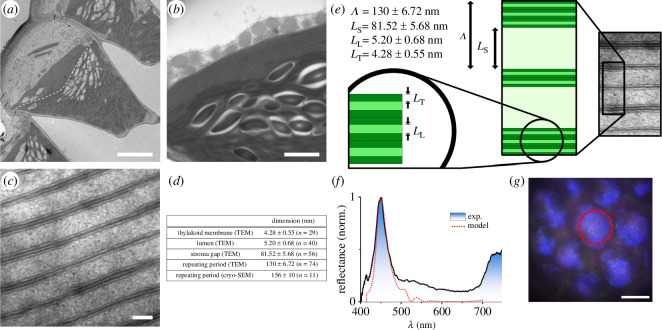
Bizonoplasts (*a*) found in the epidermis of the leaves of *S. erythropus* contain very organized periodic multi-layers (*b,c*) and conventional irregular thylakoid tissues, separated by a dashed line in (*a*). These SEM observations allow us to measure the dimensions of these structures (*d*). The period Λ of the multi-layer comprises thylakoid membranes of thickness $L_{\text{T}}$, lumen layers of thickness $L_{\text{L}}$ and stromal layers of thickness $L_{\text{S}}$. Incident light reflects on this multi-layer in the blue, as evidenced by spectrophotometry and simulations (*f*) as well as visualized through light microscopy (*g*). Scale bars: 5 μm (*a*), 0.5 μm (*b*), 100 nm (*c*) and 10 μm (*g*). These figures were reproduced from [95], with permission from the Royal Society.

The blue iridescent epidermal chloroplasts occurring in some plant leaves, such as those in shade-dwelling *Begonia* spp. and *Selaginella erythropus*, display intricate multi-layers that significantly enhance light absorption [35,95–97]. Chloroplasts, crucial plant organelles facilitating photosynthesis by absorbing incident light via chlorophyll, play a pivotal role in converting light energy into biochemical energy as adenosine triphosphate (ATP) and nicotinamide adenine dinucleotide phosphate (NADPH). Particularly, the initial light-dependent phase of photosynthesis takes place within the absorbing thylakoid tissues of chloroplasts. Two distinct types of chloroplasts are of interest concerning photonics and enhanced light absorption: iridoplasts and bizonoplasts [98]. Iridoplasts, exclusive to some plants such as the leaves of *Begonia*, possess photonic structures consisting of periodic multi-layers of thylakoid tissues. Conversely, bizonoplasts have been identified in a single plant species, *S. erythropus* (figure 11) [99,100]. They look like a mix of conventional thylakoid tissue present in typical irregular chloroplasts across many plants and very organized iridoplasts. The unique ordered photonic structures of iridoplasts and bizonoplasts result in enhanced light absorption in the green part of the electromagnetic spectrum due to the slow-light effect occurring at the red edge of the photonic bandgap of the multi-layered structures. This increased absorption aligns with the incident light environment of these canopy-adapted plants. It leads to an enhanced quantum yield in low-light conditions, bolstering photosynthesis when compared with normal chloroplasts [35,95–97].

**Figure 12.**
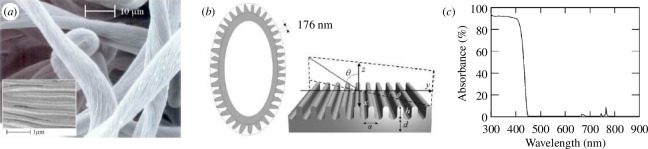
Protection against UV is a crucial attribute for edelweiss flowers (*L. nivale*) as their silver-white bracts are exposed to Sun radiation. These bracts are enveloped by a downy layer consisting of an intersecting pattern of transparent filaments (*a*) with diameters of *ca* 10 μm, as observed by SEM. These filaments display some corrugation (*a*, inset) with a period of *ca* 180 nm. The total reflectance of a bract at normal incidence is consistently high throughout the visible range but sharply diminishes to zero in the near UV. In the simulation models, the curvature of the hollow filament can be neglected (*b*). The simulated absorbance spectrum through the corrugated-slab model for transverse electric light polarization exhibits a corresponding high intensity in the 300–450 nm range (*c*). These figures were reproduced from [101], with permission from the American Physical Society.

In the ethereal heights of the Alps, the edelweiss flower, *Leontopodium nivale*, unveils a captivating defence mechanism against intense UV radiation. Located within the woolly cover layer of its bracts (namely, the downy-white ‘petals’ of the edelweiss that are specialized leaves), an intersecting pattern of transparent filaments, displaying some faint iridescence, can be observed by microscopy (figure 12). These filaments exhibit variations in the diameter and morphology of their cross-sections. They are hollow with parallel corrugations running along the main axis of the filaments with a period of *ca* 180 nm. The spectral reflectance is rather low from 300 to 400 nm and abruptly increases around 400 nm to form a plateau at *ca* 65%. This optical behaviour corresponds to some strong absorption in the near UV range. This intricate filamentary architecture, akin to a two-dimensional corrugated dielectric slab, emerges as a UV-selective waveguide coupling device. Fano resonances within these filaments facilitate the transfer of incident UV waves. The filaments, characterized by a broadband angular response, act as conduits for UV photon energy, efficiently dissipating it along the hollow guides, as the filament materials absorb UV. This ingenious strategy protects the delicate cellular tissue beneath.

## 3. Infrared absorbers inspired by natural photonic structures

The underlying mechanisms of various natural structures have been elucidated thus far, as presented in the previous section. If their potential for energy harvesting remains largely untapped, several devices have been suggested through a bioinspiration approach [14,15,17]. In addition to their eco-friendly and sustainable materials composition and fabrication, these natural structures present other benefits concerning current alternatives such as their thinness and lightness. They can improve the energy yield of PV cells and solar panels [29,47,102], passive radiative cooling [37,103–106], photocatalysis [45,107–109] or even the efficiency of electromagnetic camouflage, and the capture of stray light in telescopes. These bioinspired applications are made of different materials and may require a different range of wavelengths as well as even higher light-intensity absorption. That is why their design often involves additional optimization, resulting in structures exhibiting a similar but not exact morphology with different dimensions. Changing the wavelength range implies the adjustment of the structure dimensions due to the scalability of Maxwell’s equations.

### 3.1. Bioinspired antireflective coatings: from moth-eye and cicada-wing templates to functional applications

As described in §2.1, insects employ antireflective features as crucial characteristics in their crypsis strategy. The nipple arrays found on some of their eye and wing surfaces have been replicated to create bioinspired antireflective coatings applicable across various uses, including solar panels, antiglare glasses, screens, light-sensitive detectors, telescopes, thermochromic smart windows and camera lenses [14,69,102,110–117].

**Figure 13.**
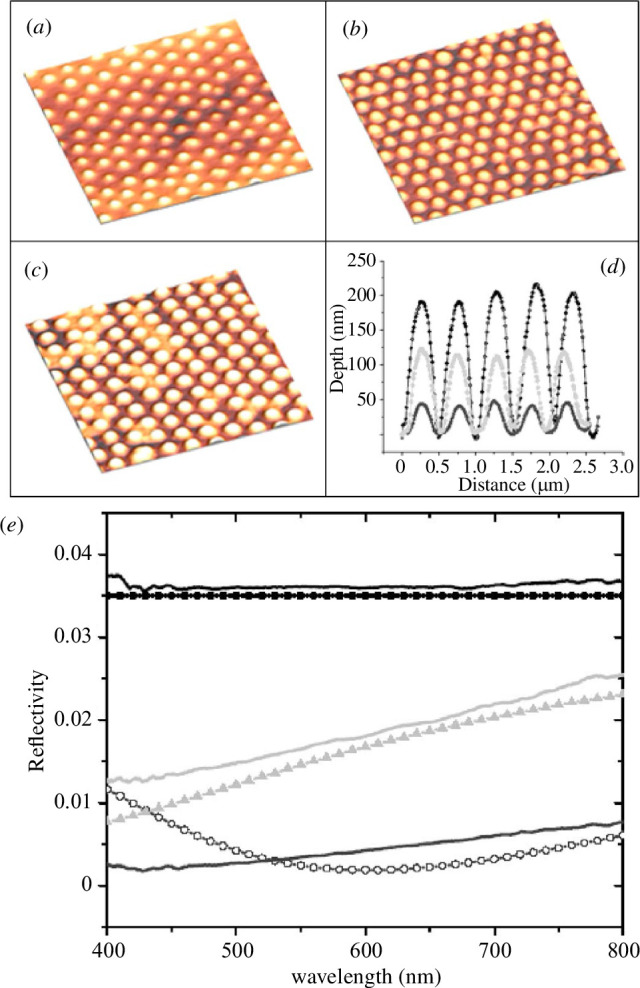
Nipple array structures observed on moth eye were replicated using 360 nm size silica particles, observed in (*a–c*) by atomic force microscopy (AFM) without etching (*a*) as well as with 20 s (*b*) and 45 s (*c*) reactive ion etching (RIE) etching times. This etching process gave rise to different profiles (*d*). This bioinspired structuring gives rise to a significant reduction in light reflection at normal incidence (*e*). Solid and dotted curves are respectively experimental and simulated reflectance spectra with a flat unstructured poly(ethoxylated trimethylolpropane triacrylate) (PETPTA) surface (black curves), 110 nm size hemispherical caps (light grey curves) fabricated with 20 s RIE etching, and 180 nm size hemispherical caps (dark grey curves) fabricated with 45 s RIE etching. These figures were reproduced from [110], with permission from AIP Publishing.

Bottom-up nanofabrication approaches such as self-assembled spherical nanoparticles in non-close-packed hexagonal arrays have been used to mimic moth-eye structures (figure 13) [110]. Simulations indicated that non-close-packed structures result in lower reflectance at wavelengths longer than the nipple interdistance, affirming the suitability of the natural moth-eye design for highly efficient antireflective devices. Spin-coating deposition of colloidal suspensions of silica particles (360 nm in diameter) and their shear alignment allowed fabrication of a template that served as a mould for casting some polydimethylsiloxane (PDMS). This PDMS mould was subsequently pressed onto an ethoxylated trimethylolpropane triacrylate (ETPTA) [110] or a perfluoroacrylate polymer [118] layer lying on a glass substrate (figure 13*a*–*d*). These monomer films were then polymerized with a pulsed UV light curing system. Such biomimetic structures demonstrated outstanding low reflectance of less than 0.5% across the visible spectrum (figure 13*e*) [110,118]. Similarly, such spin-coated monolayer silica colloids were utilized as a mask in a reactive ion etching (RIE) process of silicon wafers with SF_6_ [111] and of gallium antimonide (GaSb) substrates with Cl_2_ [102], reducing reflectance to less than 5% in the visible-near-IR range (concerning *ca* 40% for unstructured wafers) [102,111]. Such biomimetic structures appeared easy to fabricate on solar and TPV cells.

**Figure 14.**
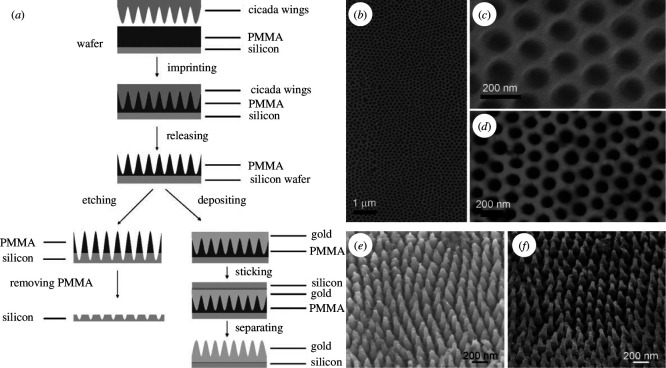
Nipple array structures were replicated in gold from cicada wings by NIL (*a*) [69]. A negative replica was also fabricated in silicon (*a*). The process involved using cicada wings as a natural stamp, giving rise to a PMMA negative replica observed by SEM (*b,c*) and AFM (*d*). Depositing gold on this PMMA mould allowed the fabrication of a perfect replica of the gold hexagonal nanopillars (*e*) in comparison with the natural-wing structure (*f*). These figures were reproduced from [69], with permission from John Wiley and Sons.

Similarly, top-down synthesis techniques such as nanoimprint lithography (NIL) were employed to develop efficient and cost-effective antireflective coatings inspired by nature. For instance, cicada wings were directly utilized as natural stamps, leveraging the wing chitinous material with commendable thermomechanical characteristics (figure 14*a*) [69,112]. This material can indeed be heated up to 200°C without any damage. After heating, a poly(methyl methacrylate) (PMMA) film may be pressed onto the biological template (figure 14*b*–*d*) [69]. The negative replica was transferred to a silicon substrate using the array of nanowells in PMMA as a mask for RIE. Upon removal of PMMA, the resulting patterned surface demonstrated antireflective properties, evident from its dark visual appearance [69]. If the structured PMMA film was employed as a mould for gold thermodeposition instead of a mask for RIE, a perfect replica of gold hexagonal nanopillars was produced (figure 14*e*,*f*) [69]. Alternatively, a PMMA replica was fabricated through a modified procedure [112]: a first negative replica was obtained from the thermodeposition of gold onto the natural photonic structures of the wings. It was used as a mould to cast PMMA, which was subsequently peeled off [112]. The antireflective capability of the replicated PMMA film was notable. The PMMA film’s reflectance was found to be decreased from approximately 6% to about 2% across the visible-near-IR range due to the unique nipple array [112].

### 3.2. Advances in bioinspired solar light harvesting: beyond petals, leaves and butterfly wings

**Figure 15.**
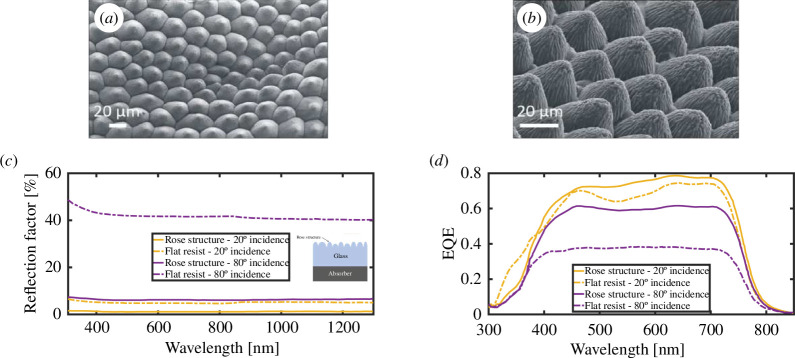
The microstructures occurring on the integument of rose petals were replicated in PMMA for application onto solar cells (*a,b*). They are observed here by SEM. The reflection factors from a glass substrate, featuring a black absorber on the rear and the replicated rose structure on the front side with light incident at angles of 20° and 80° (solid lines) are significantly lower than flat unstructured references (dashed-dotted lines) (*c*). Consequently, the corresponding external quantum efficiencies (EQE) of the structured thin-film organic solar cell are higher than those of the flat references (*d*). These figures were reproduced from [119] (*a,b*), License CC-BY-4.0. Data for (*c,d*) were sourced from [34].

The photonic structures found in the petals and leaves occurring in the integuments of certain plants have informed the development of improved bioinspired light-absorbing structures (figure 15*a*,*b*) [34,88,107,108,119,120]. From enhancing the efficiency of organic solar cells to improving photocatalysis and contributing to artificial photosynthesis systems, these bioinspired designs continue to pave the way for sustainable and innovative energy solutions.

For instance, the microstructures of the epidermal cells of some rose species inspired a polymer thin film replica that was integrated into a solar cell [34]. The biomimetic coating demonstrated a significant reduction in reflectance over the entire spectral range, particularly at grazing incidence, with a remarkable 13 and 44% increase in the solar cell’s short-circuit current at normal and grazing incidence, respectively (figure 15*c*,*d*). These properties are of high interest for solar cell efficiency with respect to the Sun’s movement throughout the day. The dome profile of the micropapillae on the petal surface was demonstrated to play the role of microlenses, lengthening the optical path of light rays within the plant integuments. This dual functionality of efficient antireflection and light trapping is crucial for enhancing the performance of thin-film organic solar cells, addressing issues of low optical absorption and spectral drops due to Fabry–Pérot interference.

While the petals of roses offer a compelling template for biomimetic light harvesting, the sophistication of plant leaves provides an even more complex blueprint. The thin and soft leaves of *Vallisneria* spp., aquatic grass plants, also known as eelgrass are a very informative study case for bioinspiration [107]. The hierarchical architecture of these leaves includes lens-like epidermal cells, a so-called palisade parenchyma functioning as optical waveguides, and a spongy disordered layer with intertwined veins giving rise to optical multi-scattering and extending the optical path length. Utilizing a sol–gel method, a silica and titania mimic of these leaves was templated for photocatalytic application [107]. The resulting Ti–Si catalyst exhibited a threefold higher rate constant for the degradation reaction of methylene blue exposed to UV compared with a commercial TiO_2_ catalyst. The macroporosity and enhanced light-scattering properties of *Vallisneria* leaf structure made it an ideal template for photocatalysis, showcasing the potential for biomimicry in advancing solar-driven environmental applications.

**Figure 16.**
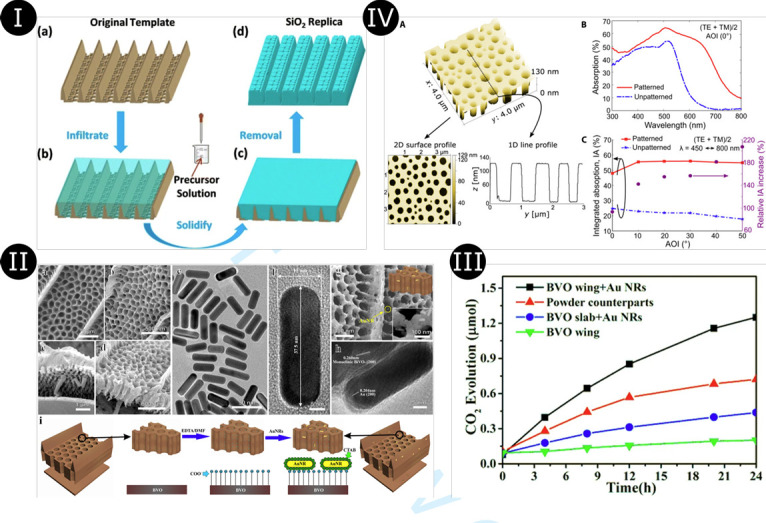
(*I*) SiO_2_ negative replica of the black wing scales of *Trogonoptera brookiana* butterfly (*a*) was synthesized through a sol–gel method [29]. The scales were infiltrated with a precursor solution (*b*) that was heated for solidification (*c*). The scales were removed by etching (*d*), leaving a negative replica. (*II*) A sol–gel method was also used to fabricate a bismuth vanadate (BVO) replica (*b,d*) of the wing scales of *Papilio nireus* (*a,c*) [45]. Gold nanoantennas (abbreviated ‘Au NR’) (*e,f*) were loaded into the BVO wing scales (*g,h*). Insets in (*g*) are a simplified sketch (top) and a higher-magnification electron micrograph (bottom). The fabrication process from the butterfly wing scale to the Au NR-loaded BVO hybrid photonic–plasmonic structure is summarized in (*i*). (*III*) This hybrid structure gave rise to the best photocatalytic activity [45], as demonstrated by the CO_2_ evolution of isopropyl alcohol (IPA) degradation as a function of illumination time for the Au NR-loaded BVO hybrid photonic–plasmonic structure (‘BVO wing + Au NRs’), unstructured BVO powder (‘Powder counterparts’), unstructured BVO slab with Au NRs (‘BVO slab + Au NRs’), and bio-templated BVO photonic structure (‘BVO wing’). (IV) A nanostructured hydrogenated amorphous silicon (a-Si:H) film inspired by the wing scales of *Pachliopta aristolochiae* (*a*) gave rise to enhanced light harvesting at normal incidence (*b*) and with non-zero incidence angles (*c*) [47]. These figures were reproduced from [29] (*I*), [45] (*II,III*) and [47] (*IV*), Licenses CC-BY-4.0, CC-BY and CC-BY-NC, respectively.

As one could have expected, butterfly wings have naturally inspired the design of visible and IR light absorbers such as a SiO_2_ negative replica of the black wing scales of the *Trogonoptera brookiana* butterfly that exhibits enhanced light trapping properties (figure 16(i)) [29]. The solar energy loss of this replica, namely, the integrated solar light intensity reflected by the replica in the 400–900 nm range, was found to be 22.6% of the one of a SiO_2_ flat surface. Similarly, a hybrid photonic–plasmonic structure was fabricated by bio-templating the black forewings of *Troides helena* [30]. Silver spherical nanoparticles with various diameters (10, 20, 40, 60 and 80 nm) were deposited on the wings before the chitin structure was carbonized. An enhanced absorption was measured and simulated in the near- and mid-IR ranges. It results from the plasmon resonance of the silver nanoparticles and the coherent coupling among adjacent nanoparticles within the photonic architecture of *T. helena*. Such a hybrid photonic–plasmonic architecture was designed for photocatalytic applications while taking inspiration from *P. nireus* (figure 16(ii)) [45]. It consisted of gold nanoantennas located on a bismuth vanadate (BVO) photocatalytic unit with the architecture of *P. nireus* black wings. This architecture was fabricated through a sol–gel method. Both experiments and simulations demonstrated the enhanced photocatalytic activity arising from the 25% increase in light harvesting within the 700–1200 nm range and the 3.5-fold enhancement of the electric-field intensity of localized surface plasmons (figure 16(iii)) [45]. Whereas these three artificial structures were fabricated by bottom-up methods, a nanostructured absorber film with disordered holes was synthesized as a mimic of the disordered black wing scales of the *P. aristolochiae* butterfly (figure 16(vi)) [47]. Using phase separation of a two-polymer mixture, a hydrogenated amorphous silicon (a-Si:H) film was patterned for PV applications. The structure exhibited a relative integrated absorption over the range 450–800 nm of 93 and 207%, with a 0° and a 50° incidence angle with respect to the normal to the film surface, respectively [47].

## 4. Conclusions

Photonic structures occurring in the integuments of living organisms such as arthropods, birds and plants are very sophisticated optical devices that give rise to various striking optical effects, including UV, visible and IR radiation management and absorption enhancement. They occur in biological tissues encompassing butterfly wings, beetle elytra, bird feathers, spider cuticle, viper skin, as well as plant leaves and petals. These phenomena are often crucial for the survival of animal and plant species. This review article also showcased the promising potential of bioinspiration in the field of energy capture and conversion. Exploiting light trapping, impedance matching or antireflection observed in natural structures is indeed highly interesting, given the development of bioinspired energy-efficient applications such as PV and TPV cells, TEG, artificial photosynthesis and photocatalysis. With the optimization of the efficiency of such applications, these advances inspire future research and innovation in the field of bioinspired energy solutions. Ultimately, this research paves the way for a more sustainable and environmentally conscious future by harnessing the beauty of nature’s designs to meet humanity’s energy needs.

## Data Availability

This article has no additional data.

## Footnotes

^1^
The scaling properties of Maxwell’s equations allow the dimensions of a photonic structure to be adjusted for operation at different wavelengths just by proportionally scaling the dimensions or material properties about the change in wavelength.

^2^
The absorptance A corresponds to the relative light intensity absorbed by a given medium or structure with respect to the incident intensity. It is defined as A=1−R−T, where R and T correspond to the reflectance (relative reflected light intensity) and the transmittance (relative transmitted light intensity), respectively. This quantity is bounded to 1.

^3^
Radiative cooling is the physical process through which every body emits thermal radiation, cooling down due to a loss of heat. It is described by Planck’s Law.

^4^
Gradual refractive-index matching reduces light reflection, as any incident light experiences no abrupt change in refractive index. This can be approximated by a stack of thin layers with a slightly larger (or lower) refractive index. With a sufficiently small contrast in refractive index between two adjacent layers, very low light intensity is reflected.
